# Traditional medicine practices and dimensions in the North Al-Batinah region of Oman: frankincense as a case study

**DOI:** 10.3389/fsoc.2026.1814421

**Published:** 2026-05-14

**Authors:** Hajer Harrathi, Aida Al-Qasimi, Mohamed Dridi, Nawal Al-Sheerawi, Mediha Jlassi

**Affiliations:** 1 Department of Arabic Language and Literature, Sohar University, Sohar, Oman; 2 Department of History, Sultan Qaboos University, Seeb, Oman

**Keywords:** frankincense, healing rituals, Omani society, social representations, traditional medicine

## Abstract

This study aims to explore traditional medicine in Oman as an integral part of the country's deeply rooted cultural heritage that continues to influence the present through its resilience and persistent roles. The research takes as a case study the practice of healing using *lubān* (اللّبان, *frankincense*), which has manifested as a durable and enduring therapeutic system within the preventive and curative practices of participants in the study community. This system operates within a framework of beliefs and representations that have contributed to its continuity, despite the advancements and achievements of modern medicine in Oman. The study adopted a qualitative ethnographic approach supported by semi-structured interviews and descriptive quantitative summaries, relying on field observations and a series of interviews conducted within a defined geographical context and time frame—specifically, the Governorate of North Al-Batinah, across its six *wilāyāt* (ولايات, *districts*) during the academic year 2022/2023. The research aimed to document the most prominent methods of treatment using frankincense among participants, examining its key preventive and curative forms, as reported and practiced by participants, as well as the representations that underpin its continued use. The study reached a set of findings, most notably the identification of the ecological, historical, and cultural dimensions of frankincense-based healing practices. These practices are characterized by a degree of ritualization, a blending of natural and metaphysical aspects, and a strong connection to individuals' cultural backgrounds, social upbringing, and the representations they hold about illness and treatment with frankincense as perceived within the study community.

## 1 Introduction

Traditional medicine represents a significant cultural product that human societies have developed since ancient times as part of their interaction with nature in all its forms and manifestations—especially in contexts requiring treatment and prevention of threats to individual health and general survival. Consequently, popular medical practices are deeply interwoven with the social and cultural heritage of the communities that produced them, and cannot be separated from the beliefs and representations that contributed to their continuity and stability.

While anthropologists and folklorists have differed in naming this form of traditional healing—using terms such as *al-.tibb al-sha*ʿ*bi* (الطب الشعبي, *popular medicine*), *al-.tibb al-badil* (البديل الطب, *alternative medicine*), *al-.tibb al-muwāzi* (الطب الموازي, *parallel medicine*), and *al-.tibb al-takmili* (الطب التكميلي, *complementary medicine*)—there is near-unanimous agreement on the definition provided by the World Health Organization (WHO). The WHO defines traditional medicine as

“the sum total of the knowledge, skills, and practices based on the theories, beliefs, and experiences indigenous to different cultures, whether explicable or not, used in the maintenance of health as well as in the prevention, diagnosis, improvement, or treatment of physical and mental illness.” (World Health Organization, 2013)

Despite the significance of scientific discoveries and medical advancements achieved by modern medicine, numerous field studies have demonstrated a noticeable rise in what is referred to as *alternative* or *ancient healing* approaches. As (Dabousha 2020) notes, there has been “a significant increase in the turn toward what is called alternative medicine or traditional healing” (p. 411), particularly during epidemics and pandemics. Although researchers have observed a convergence of global strategies in addressing illness and prevention due to globalization, many countries have retained

“distinct experiences in treatment and prevention shaped within their local popular culture. These experiences consist of a body of inherited knowledge, practices, perceptions, and expertise that reflect a medical model and therapeutic style passed down from ancestors to descendants.” (Harrathi, 2023b, p. 556)

In Oman—as in many countries around the world—traditional popular medicine is not limited to times of health crises or outbreaks. Real-world evidence confirms its ongoing presence alongside formal medical systems, with both traditions

“existing side by side, each holding significant importance. One is referred to as scientific medicine, relying fundamentally on experimentation and scientific research, while the other is traditional medicine, whose treatment methods are based on inherited cultural knowledge passed orally from one generation to the next.” (Saffari and Shein, 2013, p. 197)

Indeed, some field studies suggest widespread reliance on traditional medicine among Omanis. For instance, Ah. mad Al-Mukhini's (unpublished) study estimates that in 2004,

“80% of rural inhabitants in Oman would use traditional remedies before even considering visiting a doctor.” (Weber, 2011, p. 239)

It is within this context that the current study situates itself, shedding light on the healing practices involving *lubān* (اللّبان, frankincense) in Oman as a specific form of traditional medicine that has become

“a core issue recently imposing itself on social discourse and academic inquiry alike.” (Touhami and Hussein, 2018, p. 186)

This growing attention has emerged not only from researchers but also from international organizations such as the WHO, which has emphasized the need to integrate this vital domain into healthcare institutions, given both its importance and its deep entrenchment in local cultures. In line with this, the WHO has developed a strategic plan for traditional medicine aimed at

“leveraging the role of traditional medicine in achieving people-centered healthcare and promoting its safe and effective use through regulation and research.” (World Health Organization, 2013, p. 11)

The research problem arose from our observation of the study community, where two distinct therapeutic systems shape the daily healing and preventive practices of people—both during ordinary times and in the face of outbreaks and epidemics: modern medicine and traditional popular medicine, particularly the practices involving herbal and plant-based remedies, with *lubān* (اللّبان, *frankincense*) being the most prominent. While some Arab studies have attributed the prevalence of traditional medicine to

“the inadequacy of modern medical care, the high cost of medications, and, at times, their unavailability,” (Bin Arous, 2016, p. 78)

this explanation falls short of accounting for the Omani context. The Omani community demonstrates a clear inclination toward traditional practices such as h.ijāma (الحجامة, *cupping therapy*), tajbir (التجبير, *bone-setting*), ruqya (الرقية, *spiritual healing*), Qur'anic healing, and, most notably, frankincense-based remedies, despite the country's advancement in modern medical institutions. Our decision to conduct this in-depth study of frankincense healing practices and their representations in Oman was encouraged by two earlier studies, both of which confirmed the importance of frankincense in the therapeutic and preventive practices of Omanis.

The first study is Harrathi's (2023b), that examined a sample of individuals infected with the virus. It investigated

“the practices involving traditional foods, drinks, and disinfectants as part of Omani cultural heritage and how these were utilized during the COVID-19 crisis.” (Harrathi, 2023b, p. 103)

The study concluded that popular heritage holds significant therapeutic value, encompassing beliefs, concepts, and practices employed by past generations to confront epidemics and later adopted by their descendants to combat the coronavirus. It also revealed the central role of specific elements, with frankincense emerging as one of the most essential components of these traditional therapeutic practices. The second study, which took an anthropological approach, highlighted the centrality of frankincense in the daily life of Omanis. It found that frankincense practices are ritualized behaviors observed during occasions such as childbirth, marriage, and harm prevention, operating

“within a system in which all members of the community participate in what appears to be a spontaneous manner. However, closer examination of these daily practices reveals that they are governed by an underlying logic and regulated by certain rules.” (Harrathi, 2023a, p. 103)

In light of this consensus regarding the significance of frankincense, the present study aims to shed further light on frankincense healing practices as part of Oman's traditional medicine. Beginning from the concept of harm prevention, we sought to deepen the research into the preventive and therapeutic roles of frankincense, framing these roles as systems belonging to the wider structure of traditional Omani medicine. Based on this perspective, the objectives of this study are as follows:

- To identify therapeutic and preventive practices derived from Omani popular heritage that utilize frankincense.
- To explore the representations and perceptions associated with illness and frankincense-based healing, seeking to understand the reasons Omanis continue to resort to these methods despite globalization and the information revolution, both of which have transformed many aspects of Omani life, including healthcare, with modern medical centers now equipped with the latest technologies.

The broader aim of this research is to preserve an essential element of Omani intangible cultural heritage by documenting certain therapeutic methods, practices, and beliefs as key components of Omani identity. Supporting our choice of this topic is the scarcity of anthropological studies, both Omani and non-Omani, that have addressed traditional medicine in Oman. In this respect, the study is primarily concerned with the ethnographic documentation and analysis of these practices—focusing on their uses, meanings, and perceived effectiveness within their socio-cultural context—rather than the development of a formal theoretical model or the evaluation of their clinical or biomedical efficacy.

## 2 Literature review

The limited body of research can be classified into two main categories: the first focusing on traditional medicine in Oman in general, and the second dealing specifically with frankincense-based practices. Studies in the first category of research on traditional medicine in Oman can themselves be subdivided into two parts:

The first subgroup focuses on the general presence of traditional medicine in Oman and catalogs its main forms. For instance, (Al-Riyami et al. 2023) (, p. 91) study aimed to “assess the knowledge, attitudes, and practices related to traditional medicine in Oman and explore the factors that necessitate its use.” The study found that traditional medicine is widely used in Oman, with 90% of male participants in the survey reporting familiarity with its forms, and 81% believing in its effectiveness. The most recognized forms of traditional medicine among participants included herbal remedies, h.ijāma (الحجامة, *cupping*), and massage (Al-Riyami et al., 2023, p. 90).

This study also benefited from literature that describes key traditional healing practices in Oman from the perspectives of medical anthropology and medical sociology—disciplines concerned with the social, economic, environmental, and psychological factors influencing health and the spread of disease. Notably, Weber's (2011) research into Oman's medical history and its relation to the persistence of traditional medicine used fieldwork, archival sources, and unpublished materials to demonstrate that Oman's medical heritage is diverse and rich. Due to its geographic location, Oman has been influenced by Indian, Islamic-Persian-Arab, African, and Portuguese healing rituals and traditions.

(Weber 2011) also noted that medical traditions in northern and central Oman show influence from Greek medicine, while the southern Dhofar region preserves distinct local practices, shaped in part by African influence—particularly notable given Oman's historical rule over Zanzibar. The study cataloged numerous inherited healing methods, such as bone-setting (tajbir), prophetic medicine (al-t.ibb al-nabawi), honey-based therapies, cupping, and branding (al-kayy). (Weber 2011) concluded that Oman now possesses a modern healthcare system with remarkable indicators, especially compared to conditions prior to 1970. Nevertheless, evidence from local herb sellers, the author's fieldwork, and unpublished ethnographies from students and faculty at Sultan Qaboos Medical College confirms that traditional techniques and remedies—such as branding, cupping, herbal mixtures, honey, and exorcisms—remain widespread. The researchers also observed that the Omani government supports traditional medicine as part of its cultural heritage.

The second subgroup of this category addresses specific diseases commonly found in Oman and investigates how traditional medicine is used to treat them. For example, (Al-Kindi et al. 2011) focused on the use of complementary medicine among diabetes patients in the Muscat region, concluding that despite the “comprehensive development of Oman's healthcare system, the availability of modern facilities, and access to free medications, traditional medicine is still widely used” (p. 62). Al-Kindi also found that a significant number of diabetes patients resorted to traditional medicine, with most relying on herbal treatments. Other studies in this subgroup, such as (Al-Lawati and Al-Lawati 2020), have emphasized the need to document traditional Omani healing methods involving medicinal plants, since this knowledge often exists only in the oral memories of the elderly. Al-Lawati called for the preservation of these practices as key evidence of the therapeutic value of local herbs. However, the study also cautioned against potential side effects, especially since an investigation into six locally used medicinal plants concluded that

“the only difference between medicine and poison lies in the dosage” (Al-Lawati and Al-Lawati, 2020, p. 12).

The second category of studies is, however, more directly related to our research due to its focus on frankincense (lubān)—its tree, resin, and uses. This category also falls into two main branches. The first branch includes biological and laboratory-based studies investigating the medicinal properties of frankincense. One example is (Al-Yasiry and Kiczorowska 2016), who sought to verify the therapeutic benefits attributed to frankincense—a substance long used in traditional Eastern medicine for its antiseptic, anxiolytic, and anti-inflammatory effects. Their laboratory research into its chemical composition confirmed that frankincense oil has analgesic, calming, and antibacterial properties. It was shown to alleviate inflammatory conditions such as rheumatism, colitis, bronchitis, and sinusitis, while also reducing asthma risk when inhaled or consumed. Additionally, the study noted its anti-tumor and anti-cancer potential. Other relevant contributions include Western studies that treat frankincense as a key component of traditional medicine. These studies explore its biological components—including its acids, oils, and proteins—and affirm its anti-inflammatory benefits (Cerutti-Delasalle et al., 2016).

The second branch of this category includes anthropological studies, which explore the cultural and ritual significance of frankincense in ancient healing systems. In these civilizations, frankincense symbolized purity and sanctity. It was

“burned to ward off infectious diseases and evil spirits.” (Ramesh, 2014, p. 44)

We also benefited from research in the anthropology of the senses and olfaction, where frankincense is identified as

“a fragrant resin extracted from the bark of the frankincense tree.” (Ramesh, 2014, p. 46)

French historian and anthropologist (Le Guérer 2002), drawing on scientific and anthropological data, demonstrated that scents possess unique powers—especially in communication, healing, and cultural life—along with their ability to evoke and revive the past (Harrathi, 2023a, p. 14).

Despite this rich body of Arab and global literature, our review revealed a significant gap: no studies specifically address the therapeutic system of frankincense in Oman—even though many sources acknowledge the longstanding use of frankincense in healing and recognize Oman as the land of frankincense since the 16th century BCE (Harrathi, 2023a, p. 14). This observed centrality of frankincense in traditional healing and prevention among Omanis—both inherited and continuously practiced—justifies the anthropological lens of the current study. We adopted thick description based on ethnographic methods that involve

“interrogating informants and observing rituals.” (Geertz, 1998)

This was achieved through numerous interviews and extensive observations made possible by our immersion in the study community and active engagement with its people—to see what they see and feel what they feel.

## 3 Methodology

This study was based on fieldwork and semi-structured interviews. The fieldwork was conducted in the Governorate of North Al Batinah, situated along the Gulf of Oman. It comprises six *wilāyāt* (districts), with Sohar at its center. To the north are Liwa and Shinas, while to the south lie Sahm, al-Khaboura, and Al-Suwayq. While the formal study period extended from early September 2022 to late May 2023—during which field data were documented—the data collection process through both participant and non-participant observation had already begun long before, as a result of our residency in Oman and our active integration into the social life of the study community.

This study adopts a primarily qualitative ethnographic design, grounded in sustained immersion within the study community and supported by semi-structured interviews. In addition to qualitative analysis, the study incorporates descriptive quantitative summaries derived from interview data to identify recurring patterns in participants' responses. The interview protocol combined open-ended questions with structured prompts, allowing for both in-depth narrative accounts and the systematic capture of comparable responses. Interviews were conducted using a semi-structured format, and their duration ranged between one and four hours, depending on participants' availability and the depth of discussion. The interview guide was structured around key themes related to demographic characteristics, the relationship between traditional and modern medicine, and the uses and meanings of frankincense in healing practices. The tabulated data presented in this study were generated through the coding and categorization of recurrent themes, expressions, and reported practices related to frankincense use. Frequencies and percentages were then calculated to illustrate the distribution of these patterns within the sample. This combined approach enables a more comprehensive understanding of frankincense-based practices by linking ethnographic depth with structured descriptive analysis.

Regarding the study sample, a combination of convenience and snowball sampling strategies was employed to facilitate access to participants across the six wilāyāt (districts) of the North Al-Batinah Governorate. Initial recruitment was supported by university staff and students from the Arabic Language and Literature program at Sohar University, who assisted in identifying potential participants within their communities. This process was subsequently extended through snowball sampling, whereby participants referred the researcher to additional individuals. The sample included 280 participants from different districts and was designed to reflect diversity in age groups, educational levels, and social backgrounds, as detailed in Table 1. Efforts were made to ensure balanced representation across local contexts, including both Badaw (rural/Bedouin) and H. adhar (urban) communities, as well as across the six districts, so that no single locality disproportionately dominated the sample.

**Table 1:** Demographic characteristics of the study participants (*N* = 280).

Variable	Category	Frequency	Percentage
Gender	Male	120	42.85
	Female	160	57.15
Environment	Urban	150	53.57
	Rural	130	46.43
Age group	18–30	85	30.35
	31–50	95	33.92
	Over 50	100	35.71
Educational level	Illiterate	54	19.28
	Primary & Secondary	95	33.92
	University	86	30.71
	Postgraduate	45	16.07
Wilāyah (District)	Al-Suwayq	50	17.85
	Al-Khaboura	48	17.14
	Sahm	50	17.85
	Sohar	54	19.28
	Liwa	41	14.64
	Shinas	37	13.21
Monthly income	Less than 500 OMR	87	31.07
	Between 500 and 1,000 OMR	93	33.21
	More than 1,000 OMR	100	35.71

Although the sampling strategy does not allow for statistical generalization, it enabled the collection of rich and varied qualitative data relevant to the study objectives. In several instances, the number of individuals willing to participate exceeded the required sample size, allowing for selective inclusion while maintaining balance. No cases of refusal were recorded. Participants demonstrated a high level of engagement and willingness to share their experiences, including sensitive aspects of healing practices, under conditions of assured confidentiality.

One key limitation encountered during data collection was the concern among some participants about potential social or tribal exposure within their local environments. To address this, and with participants' consent, certain interviews were conducted away from their home districts to ensure privacy and encourage open and candid responses.

Table 1 illustrates the distribution of the sample according to several variables: gender, environment, age group, educational level, wilāyah (district), and monthly income. The diversity in gender, age, and geographic backgrounds of the participants enabled the collection of rich qualitative data regarding the knowledge, experiences, and methods related to frankincense, which individuals in the study community report using for treating illness or for preventive purposes.

Data were gathered using two primary tools: observation and interviews.

- The observation tool involved closely monitoring frankincense-related healing practices and recording observable information—especially during participant observation, wherein the researcher actively engaged in frankincense-related rituals and observed both preventive and curative applications as practiced and reported by participants in contexts of both health and illness.
- The interview tool was realized through social interaction and direct engagement between “the researcher, who receives, collects, and classifies information, and the participant, who provides it” (Ayyad, 2006, p. 128). This dynamic brought us closer to the thoughts and cultural representations of the participants, allowing us to assess the presence of frankincense in both everyday healing practices and epidemic-related contexts in Omani society, and to investigate these practices as inherited actions with deep historical roots that remain actively embedded in contemporary life, particularly in North Al-Batinah.

The collected data were analyzed using thematic content analysis of the interview material (Harrathi, 2023a, p. 107). The analytical process combined both inductive and deductive coding strategies. Initially, an inductive approach was employed to identify recurring themes emerging directly from the data, such as prevention, cure, ritual dimensions of frankincense use, and distinctions between forms of affliction (e.g., envy, jinn-related harm, and the evil eye). These themes were grounded in participants' narratives and expanded through iterative reading of the transcripts. This inductive process was subsequently complemented by a deductive analytical framework informed by concepts from medical anthropology, including social representations of illness and ritual efficacy. The integration of both approaches allowed for a coherent interpretation of the data that connects empirical observations with theoretical constructs. The unit of analysis was the full narrative discourse of each participant, which was further examined through its constituent utterances to capture both individual expressions and their broader cultural context. The selection of illustrative quotations was not arbitrary; excerpts were chosen based on their frequency of recurrence, cultural significance, and relevance to the identified themes. All interviews were originally conducted in Arabic and subsequently translated into English. The translation process was carried out and cross-checked by a bilingual Arabic–English expert to ensure both linguistic accuracy and the preservation of cultural meaning embedded in the original expressions.

This study was conducted in accordance with established ethical research standards and received ethical approval from the Sohar University's Ethics and Biodiversity Committee under the approved research protocol on Omani cultural practices. Participation in the study was voluntary, and informed consent was obtained from all participants prior to data collection. Both verbal and written consent procedures were employed, depending on the context of the interview. To ensure confidentiality and protect participants' identities, all data were anonymized at the stage of transcription and analysis. Identifying details such as names were removed, and quotations were coded using initials and general descriptors. Although age and location are reported for contextual purposes, no information allowing direct identification is disclosed. All collected data were used exclusively for academic research purposes and were securely stored on Sohar University's protected digital storage systems. Access to the data was restricted through coded authorization and was limited exclusively to members of the research team.

Note finally that this study presents certain limitations that should be acknowledged. The use of convenience and snowball sampling restricts the statistical representativeness of the sample, and therefore the findings cannot be generalized to the entire Omani population. The study is geographically limited to the North Al-Batinah Governorate, and the results should be interpreted within this specific socio-cultural context. Additionally, the reliance on self-reported data may introduce subjective bias related to participants' perceptions and experiences. Despite these limitations, the study provides valuable insights into culturally embedded healing practices within the studied communities.

## 4 Results and discussion

### 4.1 The ecological, historical, and cultural dimensions of frankincense-based healing in Oman

Before analyzing the healing practices and cultural representations associated with frankincense (*lubān*), it is necessary to first explore its underlying dimensions, as it is a therapeutic substance rich in symbolic and functional significance. Among the most prominent of these dimensions are the ecological, historical, and cultural aspects.

#### 4.1.1 The ecological dimension

The ecological approach (*al-madkhal al-ikuluji*, المدخل الإيكولوجي) refers to a holistic perspective on

“the complex, reciprocal relationships between living organisms and their environments.” (Al-Makkawi, 2007, p. 16)

These interrelations take on specific dimensions when applied to human beings—unlike other living organisms—due to the ongoing interaction between humans and the life space in which they exist. This space shapes their experiences of health and illness, including how therapeutic responses are formed—such as the use of frankincense in Omani healing, which is part of the ecological system of the region.

Frankincense is a resinous substance secreted by a tree scientifically known as

“*Boswellia sacra*. When the resin is exuded and exposed to air, it solidifies into a crystalline gum known as frankincense.” (Al-Maashani, 2008, p. 115)

While naturally occurring frankincense trees are rare in the world—found mainly in Somalia, Yemen, India, and parts of Pakistan (Encyclopedia Britannica)—sources agree that Omani frankincense, especially that found in “the Dhofar Mountains,” is among the finest in quality and has been harvested there since pre-Islamic times (Al-Hamawi, n.d., p. 60). Field observations and interview data from the study community reveal the central place of frankincense in preventive and curative practices, as evidenced by its widespread use and the high level of trust in its effectiveness. These findings suggest that the prominence of frankincense in healing practices is closely linked to ecological familiarity and long-standing environmental embeddedness within the study community, which in turn reflects the adaptation of

“social groups to their environments.” (Al-Makkawi, 2007, p. 16)

From both observation and interview records, a consensus emerges: frankincense is used deliberately and regularly for healing. One participant noted:

“*Frankincense is essential in every Omani home. No house is without it. In our home, it's necessary to burn frankincense every morning and at sunset. We also use it to treat illnesses like coughing, and even when we just feel emotionally down*.” — M.M. (48 years old), personal interview, Shinas, Tuesday, 11 October 2022.

This testimony illustrates how environmental factors shape individual attitudes toward treatment,

“affirming the idea that a person is a product of their environment—sometimes it dominates them, and at other times it shapes their inclinations.” (Dabousha, 2020, p. 415)

In this context, another interviewee linked the therapeutic role of frankincense to the cultural and ecological characteristics of their surroundings:

“*Frankincense is a must in every Omani home, whether for healing, prevention, or fragrance. We don't really know why—it's just what we learned from our fathers and grandfathers.”* — M.S. (52 years old), personal interview, Shinas, Tuesday, 11 October 2022.

These insights show how the healing behaviors of the study community are influenced by the natural and ecological features of their environment—specifically, the frankincense tree—as well as by prevailing social values and ideas. All of these are situational determinants that affect people's ability to choose among available healing alternatives, as

“such factors influence individuals' ability to select the methods most likely to achieve their health goals from among various options.” (Touhami and Hussein, 2018, p. 187)

Another participant emphasized the deep ecological connection and emotional resonance of frankincense in Omani healing:

“*Frankincense incense holds a special place in our hearts and minds. Its scent is the scent of our grandmothers, the warmth of our homes. Especially in winter, we must burn frankincense in the house—for peace of mind, for fragrance, to repel mosquitoes, and also for healing.”* — J. H.. (42 years old), personal interview, Sahm, Thursday, 20 October 2022.

These findings suggest that healing behaviors within the study community are shaped by the interaction between ecological context, cultural values, and inherited knowledge systems.

#### 4.1.2 Historical dimension

Both traditional medicine in general and frankincense-based healing in particular have deep historical roots in Oman and other cultures. Traditional medicine is considered among

“the oldest bodies of knowledge pursued by humankind.” (Qayed and Sabti, 2016, p. 122)

Frankincense is one of the oldest therapeutic substances used by Omanis, having served as a medium of exchange and cultural interaction with numerous ancient civilizations since the early centuries CE, particularly through trade. The frankincense trade was historically linked to the Incense Route—a trade corridor connecting India, the Arabian Peninsula, and Egypt. One of the key hubs was Samharam Port, known for exporting frankincense to the ancient and medieval worlds as early as the fifth century BCE. According to historical accounts, some orientalists interested in ancient history narrate that the ship of Prophet Solomon docked at Lake Samharam, where jars filled with frankincense rolled down from the mountain and landed in the ship before it sailed away carrying the resin. Similarly, the Pharaohs of Egypt imported large quantities of frankincense from Dhofar as offerings to their deities.

Historical sources also mention that

“Queen Hatshepsut of Egypt (circa 1,500 BCE) imported sacred frankincense from the Land of Punt, identified with Dhofar in Oman.” (Al-Bousaidi, 2017)

Due to this historical centrality, frankincense became a

“hallmark of ancient trade routes, and the caravan roads it traversed became known as the Incense Route or Frankincense Route, which passed through many prominent cities in Dhofar, Oman.” (Harrathi, 2023a, p. 111)

In recognition of its historical significance, the Frankincense Route was inscribed on the UNESCO World Heritage List in the year 2000 (National Omani Committee for Education, Culture and Science, n.d.).

The therapeutic dimension of frankincense is also apparent in this historical context. Sources affirm that frankincense has been

“a highly valued trade commodity in North Africa and Arab civilizations for at least 5,000 years. It was commonly burned to reduce infectious diseases and to repel evil spirits.” (Ramesh, 2014, p. 44)

This enduring use of frankincense can be explained through the lens of the WHO's framework, which attributes continued reliance on traditional medicine to

“deep-rooted cultural and historical influences, where traditional healthcare systems are well-established and enduring.” (World Health Organization, 2019, p. 15)

One respondent explained:

“*When I burn frankincense in the house, I feel a sense of the old days—when we were young and my mother would burn it in the evenings or in winter. We use it more during the cold season because it brings warmth to the home. The scent of frankincense is beautiful. It's soothing for breathing and the chest, and it helps with phlegm.”* — ʿA. Q. (54 years old), personal interview, Al-Suwayq, Thursday, 2 March 2023.

#### 4.1.3 Cultural dimension

The use of frankincense in healing practices in Oman is intimately connected to culture and its components, which exert a significant influence on

“health and illness and shape people's behaviors through embedded behavioral directives.” (Bin Arous, 2016, p. 85)

Among the most influential cultural components are popular beliefs, deeply rooted in the study community, particularly those relating to how illness is explained. The most commonly cited causes of illness include the evil eye (*al-*ʿ*ayn al-sharrirah*, العين الشريرة), believed to inflict harm upon individuals; envy (*h*.*asad*, حسد), which is thought to negatively affect the envied person; and unseen entities such as jinn and devils, which are believed to interfere with people's physical health, emotions, and mental states.

Just as these folk beliefs define the causes of illness, they also suggest a variety of healing methods—many of which incorporate frankincense. One participant explained its protective use against the evil eye:

“*We always burn frankincense to guard against envy and the evil eye.”* — L. Sh. (25 years old), personal interview, Sahm, Thursday, 20 March 2023.

As revealed in both interviews and participant observation, frankincense use is embedded in a wide range of social behaviors, customs, and rituals. It is a key element in the rituals of birth, marriage, and death, and it plays a vital role in both visible illnesses—like chest pain, dental issues, and joint problems—and invisible afflictions, such as those believed to be caused by envy or spiritual harm. Another participant noted:

“*We use frankincense after childbirth for forty days, at weddings with oud (incense), and anytime someone is sick—we burn it in the home every day before Maghrib prayer. We also use it to repel mosquitoes, for a good smell that helps us sleep peacefully, especially at night. Elderly women must incense their clothes daily, and we use it for children to protect them from the evil eye and envy.”* — Z. Sh. (56 years old), personal interview, Sohar, Monday, 3 October 2022.

From these rich cultural expressions, one can discern the mythological dimensions of frankincense as a sacred substance—a status emphasized in historical sources. (Al-Qabti 1992, p. 166) explained frankincense's sanctity in terms of “the purity of the tree it is harvested from, and its power to ward off impure, harmful spirits that corrupt the intentions of sorcerers.” Oral traditions in Oman also preserve beliefs about the protective powers of the frankincense tree, which is thought to safeguard the souls of ancestors. When burned, its resin becomes a mediating substance between the living and the dead (Al-Hajri, 2018). In addition to this, the mythology of frankincense includes its purifying and healing properties, which is why it was employed by the ancient Egyptians. Some individuals in the study community cannot imagine a day without it. As one participant said:

“*We love the scent of frankincense. We're used to it daily, especially around sunset.”* — A.B. (28 years old), personal interview, Sahm, Sunday, 5 March 2023.

For others, frankincense-based healing is considered a cultural inheritance that must be preserved and passed on:

“*I raise my children to love the scent of frankincense—it's the scent of our fathers and grandfathers.”* — L.N. (62 years old), personal interview, Sohar, Sunday, 19 March 2023.

### 4.2 The reality of frankincense-based healing: forms and representations in Omani daily life

We began the interviews with a question related to participants' behavior when experiencing a health discomfort. Their responses were recorded and are summarized below:

The interpretation of these findings is informed by an anthropological perspective, particularly approaches concerned with the cultural beliefs and social practices that shape understandings of health and illness (Bin Arous, 2003). More specifically, the analysis draws on insights from medical anthropology, a field that examines health and illness as social phenomena, including how individuals interpret the body, the symbolic systems through which it is represented, and the rituals that contribute to healing processes (Foster, 1978; Boughdiri, 2017). This perspective also emphasizes the culturally embedded elements involved in the perception, diagnosis, and treatment of illness (Lewis, 1986), allowing for a deeper understanding of frankincense-based practices within their socio-cultural context.

#### 4.2.1 Forms of frankincense-based healing

The results presented in Table 2 indicate that a significant majority of participants (87.14%) reported initiating treatment through inherited healing practices rather than immediately consulting a medical doctor. These findings further suggest that recourse to traditional remedies constitutes a primary and culturally grounded response to illness within the study community, rather than a secondary or alternative option. This pattern can be interpreted through the lens of what medical anthropology conceptualizes as *medical pluralism*, whereby multiple therapeutic systems coexist within the same social context. Rather than reflecting a lack of access to biomedical care, this tendency indicates that inherited healing practices are embedded within a coherent cultural framework that links illness, prevention, and treatment to shared systems of meaning.

**Table 2:** Distribution of the sample according to whether they begin with a doctor visit or with inherited healing practices.

Question	Response	Frequency	Percentage
In case of feeling unwell, what do you prioritize: seeing a doctor or starting with some inherited Healing practices?	Start with a doctor visit	36	12.85
	Start with inherited healing practices	244	87.14

Percentages are calculated based on the total sample (*N* = 280). Multiple responses were allowed.

While (Harrathi 2023a, p. 113) highlights the strong presence of frankincense in Omani households and the diversity of its uses—particularly noting that individuals often “purchase a stock that lasts them an entire year when they travel to Dhofar, the natural source of frankincense, in order to obtain the highest quality”—the present study focuses specifically on the therapeutic dimension of these practices. It seeks to examine the various ways in which frankincense is used for healing purposes, as distinct from its roles in fragrance or general ritual use. The interview data revealed multiple forms of frankincense-based healing practices, which are summarized in the following table (see Table 3).

**Table 3:** Distribution of respondents by type of frankincense-based healing practice.

Form of practice	Males	%of males	Females	% of females	Total	Overall %
Drinking frankincense-infused water (after soaking overnight)	82	29.28	102	36.42	184	65.71
Sprinkling or distilling frankincense water on body parts	57	20.35	60	21.42	117	41.78
Rubbing, massaging, or applying frankincense oil	66	23.57	105	37.50	171	61.57
Chewing frankincense resin	42	15.00	74	26.42	116	41.42
Burning frankincense for incense (fumigation)	115	41.07	158	56.42	273	96.50

Percentages are calculated based on the total sample (*N* = 280). Multiple responses were allowed.

The table above reveals the diversity of frankincense-related practices used for either therapeutic or preventive purposes. Based on interviews and participant observation, burning frankincense for fumigation ranked highest among the practices, with 96.5% of respondents reporting it, followed by drinking frankincense-infused water (65.71%). These findings suggest that this diversity of practices does not indicate fragmentation but rather reflects the flexibility of the therapeutic system and its capacity to adapt to different types of illness and associated cultural interpretations. The predominance of fumigation (96.5%) may indicate that healing is not limited to the physical dimension but extends into the symbolic and ritual domain. In this sense, these practices can be understood in light of anthropological perspectives on *symbolic efficacy*, whereby ritual actions contribute to healing through culturally meaningful processes that shape perception, emotion, and bodily experience.

More specifically, the act of burning frankincense is reported to serve two purposes. One is understood as therapeutic by participants, particularly in cases of envy (h. asad) or spiritual affliction (mass); the other is preventive, offering protection against natural threats like viruses, fungi, and insects—especially during the winter season—as well as unseen harms, such as those linked to spiritual disturbances. As for soaking frankincense resin in water and drinking it, this practice is reported by participants as being associated with the management of asthma and other respiratory symptoms. One participant noted:

“*Frankincense—especially lubān dhakar (male frankincense)—is very good for asthma.”* — L.N. (62 years old), personal interview, Sohar, Sunday, 26 March 2023.

Others emphasized that frankincense water was believed to have boosted immunity and alleviated the inflammation and ulcer he had in his stomach. One participant shared:

“*My father's regular consumption of frankincense water boosted his immunity and healed the inflammation and ulcer he had in his stomach.”* — L.N. (62 years old), personal interview, Al-Khaboura, Tuesday, 17 January 2023.

Several participants who had contracted COVID-19 reported using male frankincense (lubān dhakar) as an immune booster, which they thought to enhance their ability to cope with the infection. As a result, soaking frankincense and drinking its water became a common practice during the COVID-19 pandemic. Another common practice is distillation or soaking frankincense for topical spraying, mentioned by 41.78% of participants. This method is especially associated with skin and hair conditions. One respondent explained:

“*Spraying frankincense water is believed to help reduce fungi and bacteria that cause dandruff and other hair-related diseases.”*

— S.Q. (37 years old), personal interview, Shinas, Wednesday, 25 January 2023.

Another participant described its benefits this way:

“*Frankincense is perceived to help keep hair and the scalp hydrated and to contribute to the treatment of fungal infections.”*

— L.M. (28 years old), personal interview, Sahm, Thursday, 24 December 2022.

Among some respondents from Bedouin communities, the use of frankincense water extends beyond humans to animals, particularly as a means reported by participants to repel flying insects:

“*Frankincense is used to drive away flying insects like mosquitoes and flies.”* — W.D. (22 years old), personal interview, Al-Khaboura, Tuesday, 22 November 2022.

As part of the healing practices identified in the collected data is the application and massage with frankincense oil, reported by 61.57% of the study population. For some, it is used preventively, while for others, it is applied for therapeutic purposes. One of the most common uses recorded was in the treatment of joint inflammation. A participant explained:

“*Frankincense oil is perceived as effective for massaging the body—it is believed to help relieve pain and swelling and provide comfort.”*

— H. A. (45 years old), personal interview, Shinas, Wednesday, 25 January 2023.

Another participant noted:

“*When I get tired from sitting for long hours in my shop, I massage with frankincense oil. It helps ease muscle tension and relieve joint pain.”*

— N.M. (38 years old), personal interview, Al-Suwayq, Sunday, 6 November 2022.

In addition to massage and topical application, frankincense oil is also mixed with body and hair cleansing products as reported by participants for use in managing fungal infections, especially on the scalp. One student stated:

“*I apply frankincense oil directly to my hair. Sometimes I add a few drops to shampoo or conditioner.”* — H.B. (26 years old), personal interview, Sahm, Sunday, 24 November 2022.

Although the practice of chewing frankincense for healing purposes was mentioned less frequently than other methods, interviews indicated that nearly all participants had tried it and many continue to do so. Participants explained that they chew frankincense for both its pleasant taste and its oral health benefits, including its perceived ability to help combat bacteria, fungi, gum problems, and bad breath. One elderly participant, referred to affectionately as *Umm Ahmad*, remarked:

“*We recommend chewing frankincense when someone has bad breath, because frankincense is considered beneficial for oral and dental health.”* — M.A. (38 years old), personal interview, Al-Suwayq, Sunday, 6 November 2022.

It is important to highlight that these frankincense healing practices are not limited to those with lower levels of education or social status. In fact, they are shared across all social categories, from the illiterate to those with advanced degrees—though differences exist in the type of frankincense used or in the frequency of its use. These findings were consistent across most interviews, regardless of the participants' demographic distribution (see Table 1). Moreover, the use of frankincense practices extended beyond individual behavior to institutional settings. During the COVID-19 pandemic, it was observed that frankincense was burned in hospitals, commercial shops, educational institutions, and other workspaces

“as part of broader policies aimed at reducing infection and controlling viral spread.” (Harrathi, 2023a, p. 113)

#### 4.2.2 Frankincense healing practices between the natural and the metaphysical dimensions

Guided by insights from cultural studies, which emphasize that each culture possesses

“its own interpretive frameworks and distinct perceptions that shape how illness is defined and how individuals respond to it—whether by seeking out a doctor or turning to other sources,” (Dhakkar, 2016, p. 76)

we undertook a focused examination of our recorded observations and interviews. This analysis revealed that, within the study community, frankincense practices operate across two distinct domains: the domain of natural medicine (*al-.tibb al-.tabi*ʿ*i*), and the domain of metaphysical or spiritual medicine (*al-.tibb al-ghaybi*). This duality reflects what medical anthropology describes as *explanatory models of illness*, in which individuals and communities construct meanings of illness that integrate both biological and symbolic dimensions. Such results highlight the coexistence of multiple explanatory frameworks within the same cultural system. As (Kleinman 1980) argues, illness is not understood solely through biomedical explanations but through culturally embedded interpretations that define its causes and appropriate forms of treatment. Within this framework, we sought to document the perceived effectiveness of frankincense in both domains from the perspective of the study participants, and present our findings in two separate tables: one for natural uses, and another for metaphysical/spiritual applications.

##### 4.2.2.1 Effectiveness of frankincense healing practices in the natural dimension

The percentages presented in Table 4 reveal the widespread use of frankincense-based practices as reported by participants for addressing biological, organic ailments—illnesses that result from dysfunctions in specific body organs or harm caused by visible natural agents such as viruses, insects, or fungi. These findings indicate that the widespread use of frankincense-based practices reflects shared understandings of illness and treatment within the study community. Frankincense appears prominently in practices described by participants for managing asthma, respiratory conditions, digestive issues, and stomach ulcers. This prevalence falls within the larger domain of herbal and natural remedies, a category of traditional treatment that

**Table 4:** Distribution of respondents based on their belief in the effectiveness of frankincense-based healing practices (Natural domain).

Perceived effectiveness	Males	% of males	Females	% of females	Total	Overall %
Treatment of asthma and respiratory conditions	100	35.70	155	55.35	255	91.50
Treatment of digestive issues and stomach ulcers	60	21.42	80	28.50	140	50.00
Treatment of joint and bone inflammation	20	7.14	50	17.80	70	25.00
Treatment of oral, gum, and dental problems	53	18.92	85	30.35	138	49.28
Treatment of skin conditions	57	20.35	150	53.57	207	73.90

Percentages are calculated based on the total sample (*N* = 280). Multiple responses were allowed.

“has begun to move toward formalization and institutional recognition by health authorities and organizations.” (Abou-Taya and Al-Nuaimat, 2017, p. 122)

According to the data gathered from the study population, many interviewees reported a preference for frankincense, often attributing this to its perceived lack of side effects, in contrast to some pharmaceutical drugs that might treat one condition while harming another part of the body. In this context, frankincense-based healing is best classified as complementary medicine, which the WHO recognizes as

“a widely practiced form of care across most countries in the world.” (World Health Organization, 2019, p. 15)

Within this form of natural traditional medicine, frankincense is reported by participants as being used both preventively and therapeutically. Field data show that over 91% of participants described frankincense as effective in treating asthma and respiratory issues. In fact, many cited books, media programs, and research studies that have supported these therapeutic claims. Others referred to their own experiences in managing respiratory conditions using frankincense. As one participant described:

“*During the days of COVID, we burned frankincense every day, soaked it in water, and drank it—especially when the lungs became weak and the coughing worsened.”* — Umm Abdullah (67 years old), personal interview, Sohar, Sunday, 1 January 2023.

The data also indicate that over 70% of the community perceived frankincense oil and water as effective for managing skin conditions, although with notable gender differences: 53.57% of women reported this use compared to only 20.35% of men. This discrepancy may be attributed to the association of these practices with cosmetic care, as many female participants mentioned the collagen-rich properties of frankincense as beneficial for hair and skin beauty.

Practices associated with the management of joint and bone inflammation also received a significant acknowledgment rate (25%). One woman explained:

“*Our house is never without frankincense—especially* *.hawjari frankincense. We burn it for envy and apply it directly to joints when there is pain.”* — S.A. (38 years old), personal interview, Sahm, Sunday, 8 January 2023.

The remaining percentages are relatively balanced between the reported use of frankincense for the treatment of digestive issues, 50%, and the reported use for addressing oral, gum, and dental problems, 49.28%. It is important to note that frankincense healing practices in the natural dimension are also believed by participants as serving preventive purposes. One participant elaborated:

“*During the days of COVID and widespread infection, we used to take out the bedding and place it over a small stand (dubāyah) and fumigate all the components of the bed twice daily—morning and evening—especially after infection. As a preventative measure, we burn frankincense every day after Maghrib prayer. It's part of our routine, and it's also effective for disinfection.”* — N.Sh. (42 years old), personal interview, Sohar, Sunday, 8 January 2023.

Another participant added:

“*Usually, we burn frankincense in the evenings, but during the COVID pandemic, we used it morning and evening. We also took honey with black seed and black pepper daily.”* — F. Sh. (67 years old), personal interview, Sohar, Sunday, 8 January 2023.

##### 4.2.2.2 Efficacy of frankincense-based healing practices within the metaphysical dimension

Following (Haidari 1999, p. 7), “anthropologists assert that folk traditions represent imitations and continuations of deeply rooted customs and beliefs embedded in the popular imagination, which cannot be easily altered or discarded.” Among the most enduring of these folk elements—particularly in Oman—are certain traditional healing and preventive practices associated with illnesses that are not easily explained through biomedical models. Throughout our fieldwork and ethnographic engagement, we encountered a significant proportion of participants from various age groups who reported belief in the presence of supernatural forces—entities thought as capable of influencing, harming, or manipulating human lives. When no biological explanation seems to account for a health condition, many individuals attribute the cause to malevolent spiritual forces such as the evil eye, envy, or jinn (spirits). Within such interpretive frameworks, the methods of treatment and protection are shaped accordingly. As a result, many individuals take proactive steps to shield themselves or their families from these harmful forces, and among the most prevalent protective rituals is the fumigation of spaces and bodies using frankincense smoke. The following table (Table 5) presents the extent to which participants believe in the efficacy of frankincense in protecting against such perceived harms.

**Table 5:** Distribution of respondents by belief in the efficacy of frankincense-based healing practices in the metaphysical dimension.

Efficacy area	Males	% of males	Females	% of females	Total	Overall %
Anxiety relief and protection from negative energy	50	17.80	120	42.85	170	60.70
Protection against and healing from envy (h. asad)	80	28.51	150	53.57	230	82.10
Protection against and healing from spirit possession (mass)	20	7.14	50	17.85	70	25.00

Percentages are calculated based on the total sample (*N* = 280). Multiple responses were allowed.

Table 5 reflects the distribution of respondents according to the type of harm they believe frankincense fumigation can heal or protect them from. The high percentages recorded in the table reveal two firmly held beliefs. These findings suggest the consolidation of two interconnected belief systems. The first concerns the existence of harmful metaphysical forces, such as witchcraft and envy (h. asad), and the necessity of taking precautionary measures to guard against them. The second relates to the perceived efficacy of frankincense in resisting and neutralizing these forces. For example, belief in frankincense's claimed ability to counteract envy reached 82%, while belief in its reported ability to dispel negative energy and bring luck and blessing reached 60.7%.

The coexistence of high levels of belief in both natural and metaphysical healing functions reflects the integration of multiple explanatory frameworks within a unified cultural system. These findings reinforce the view that there is no strict separation between the body and belief within the study context, but rather a holistic understanding of health that accommodates both material and immaterial sources of affliction.

These beliefs appear as deeply embedded convictions among frankincense users, to the extent that they are treated almost as inviolable rules. This is evidenced by frequent use of the terms “essential” (*lāzim*, mentioned 60 times) and “necessary” (*d*. *aruri*, mentioned 37 times) throughout the interview data. The commitment is not only to the presence of frankincense but also to how it is used. Its preparation and the substances added to it vary according to the type of perceived harm. Based on interviews, common elements combined with frankincense for enhanced efficacy include harmal (*Peganum harmala*), black seed (*h*. *abbat al-baraka*), salt, and black pepper. To enhance its effectiveness, many participants mentioned that they preferred that a respected person—referred to variously as *shaykh, mu.tawwi*ʿ (religious teacher), or *h*. *abbuti* (grandmother)—recite specific Qur'anic verses over the frankincense before it is used. This form is known as “recited-upon frankincense” (*lubān maqru*ʾ ʿ*alayh*).

It is important to note that the use of frankincense for protection from unseen forces is not limited to the community under study. Similar practices are widespread in Eastern and Western cultures, where beliefs about health and illness often reference metaphysical beings that instill fear in the popular imagination by threatening people's health and lives (Boughdiri, 2017, p. 503). This explains the tendency to avoid harm by seeking spiritual protection.

Sociologists and anthropologists have devoted extensive research to these practices, classifying them as part of “white magic,” which

“fulfills numerous social and psychological functions without harming others or violating societal norms.” (Abdelli, 2018, p. 135)

(Urab 2003, p. 12) argues that “scholars broadly agree that the most widespread form of white magic around the world is that which relates to healing and therapy.” As one participant put it:

“*Whenever we feel anxious or distressed, we burn frankincense.”* – Umm Ahmad, 70 years old, personal interview, Sohar, Monday, 17 March 2023.

Another explained:

“*My grandmother used to burn frankincense whenever she felt ill.”* – *L. N*., 19 years old, personal interview, Liwa, Sunday, 4 January 2023.

The analysis of these testimonies revealed near-unanimous agreement among participants that illness is often understood by them as having a metaphysical origin. Most believe that unseen forces can harm the body and mind, and that disease may result from envy, spiritual possession, or other supernatural causes, even when there are natural explanations such as bacteria or viruses. As (Phillips 2019, p. 69) notes:

“*In Omani belief, the envious eye (*ʿ*ayn al-.hasud) is capable of striking down dozens of individuals. It is believed that more than half of deaths in Oman are due to the evil eye. Thus, people must deceive or distract the eye to protect themselves from its harm.”*

Interviews also revealed the prevalence of frankincense fumigation during five key life events: birth, circumcision, marriage, death, and moving into a new home. Anthropologists describe these moments as “rites of passage” as per (Van Gennep 1909), which are universal across human societies and represent points of transition where individuals are seen as particularly vulnerable to supernatural harm. Within the studied community, it is widely believed that during such liminal moments, one must engage in rituals of protection, especially fumigation with frankincense, following clearly defined material, verbal, and performative protocols. These ritual elements will be detailed in the following table.

Table 6 reveals a set of shared rules that condition the presence of frankincense as a protective element during significant life transitions among members of the study community. The community's adherence to the use of frankincense during these transitional moments lends these practices a non-arbitrary, meaningful character, confirming their perceived efficacy and symbolic power. These rituals offer practitioners a sense of protection, enabling a temporary state of calm and security, and placing them in a favorable psychological state that helps them navigate life's crises in ways that even modern technology and science may fail to provide (Al-Mahwashi, 2020, p. 37). In all cases, these practices are characterized not only by their widespread use but also by their codification and repetition. One participant remarked:

**Table 6:** Frankincense in rites of passage.

Fumigation ritual	Material, verbal, and kinetic components
Postpartum (Nifās)	“It is our tradition to celebrate the newborn and care for the mother (the postpartum woman) by burning frankincense for forty days and nights—after sunrise and before sunset. We can even recognize houses with a newborn just by the scent.” — N. Sh. (43 years old), personal interview, Sohar, Thursday, 23 March 2023.
Circumcision	“We burn frankincense during circumcision so no one envies the child, and the wound heals quickly.” — Umm Muhammad (58 years old), personal interview, Shinas, Wednesday, 18 January 2023.
Marriage	“Just like in all large gatherings, frankincense is essential—especially to enhance the scent of ʿ*ud*. We fumigate the bridal chamber to purify the newlyweds from envy.” — K. F. (44 years old), personal interview, Luwā, Monday, 13 March 2023.
Death	“In mourning gatherings, some families burn frankincense for forty days, some only for three days. Others even fumigate the shroud with frankincense and ʿ*ud*.” — Umm Abdullah (67 years old), personal interview, Sohar, Sunday, 1 January 2023.
Moving into a new home	“Burning frankincense is essential to expel evil spirits from the house and bring blessings.” — M. A. (45 years old), personal interview, Shinas, Wednesday, 28 January 2023.

“*If your child suffers from asthma, soak frankincense grains in hot water, stir until fully dissolved, and give it to the child three times a day.”* — Umm Abdullah (67 years), personal interview, Sohar, Sunday, 1 January 2023.

These codified practices also include kinetic components accompanying the ritual of healing with frankincense—such as circling the censer (*majmar*) around the afflicted person or having the person stand above the censer while holding their clothes, allowing the smoke to fully penetrate the body. As one participant described:

“*Then the censer is passed three times around the head of the person afflicted by the evil eye.”* — A. K. (53 years), personal interview, Al-Khaboura, Sunday, 1 January 2023.

These protocols also include specific materials to be added in cases involving envy (*h*. *asad*), spirit possession (*mass*), or sorcery (*si.hr*), such as *h*. *armal* (Syrian rue), salt, black pepper, and black seed (*h*. *abbat al-baraka*). Other treatment strategies depend on whether the envious person is known. In cases where the jealous person is someone beloved—such as a mother or sister—the afflicted person is protected using a ritual described by another participant:

“*When the envious one is someone we love, like family, we ask them to chew frankincense before placing it in the censer, while reciting the two protective suras [al-Mu*ʿ*awwidhatayn], and we rotate the censer around the afflicted person from head to toe.”* — R. F. (25 years), personal interview, Liwa, Wednesday, 7 December 2022.

Another explained:

“*When the person casting the evil eye is known, we have them chew frankincense, then fumigate the afflicted person. No one envies you like the people who love you.”* — A. A. (37 years), personal interview, Shinas, Thursday, 15 December 2022.

If the envious person is known but cannot be confronted, a different approach is taken. As one woman noted:

“*We write the envious person's name and their mother's name on paper, burn it along with frankincense, and circle the censer around the afflicted person's head.”* — Gh. S. (30 years), personal interview, Shinas, Monday, 19 December 2022.

She continued:

“*We also use specific mixtures for the evil eye—every family has their own version. It can include al-shabbah (alum), h*. *armal, black seed, salt, and seven black peppercorns. Some families use al-jāwi, Indian costus (al-qust al-hindi), or ready-made ‘evil eye incense'.”* — Umm Abdullah (67 years), personal interview, Sohar, Sunday, 1 January 2023.

These material practices are accompanied by verbal ones. Some of the phrases used include:

“*In the name of God, I cast out all that harms you. May God cure you of every illness,” or “Falsehood be gone from so-and-so, son of so-and-so… If it's from the upper world, remove it; if it's from the lower world, remove it—and if you don't know where it's from, find it anyway.”* — Z. Sh. (57 years), personal interview, Sohar, Sunday, 1 January 2023.

One participant offered a detailed protocol for cases where the envious person is unknown. She said:

“*We take three small pieces of paper and write on each ‘Adam son of* *.Hawwā*ʿ*,' and on three more we write ‘.Hawwā*ʿ *daughter of Adam.' Then we mix frankincense, black pepper, and a bit of salt. We wrap this mixture into cones with the papers and burn one ‘Adam' and one ‘.Hawwā*ʿ'* cone each day for three days. The smoke must fully penetrate the afflicted's body—if it's a man, his turban is removed and placed over the censer until it's filled with smoke. He stands over the censer and holds his robe to let the smoke fill it. The censer is rotated three times around his head. Some families also rub the ashes from the cones on the afflicted.”* — Z. Sh. (57 years), personal interview, Sohar, Sunday, 1 January 2023.

When household methods prove ineffective, some turn to a spiritual intermediary:

“*If the afflicted person doesn't benefit from the usual home remedies, we consult a shaykh, who can diagnose whether the cause is natural, envy, or possession. Then he gives us water or frankincense that has had Qur'anic verses recited over it.”* — Z. Sh. (57 years), personal interview, Sohar, Sunday, 1 January 2023.

These findings suggest that the structured and repeated nature of these practices contributes to their persistence as culturally meaningful healing rituals. Despite the diversity of locations within the study area, little regional variation was observed in these practices—except in the ingredients added to frankincense. For instance, h. armal is more common in Shinas, while *al-shabbah*, salt, and black pepper are favored in Sohar. This consistency may stem from shared social representations and belief systems about both the sources of harm and the healing power of frankincense. As Moscovici reminds us, social representations constitute

“a form of shared knowledge within a community,” (Moscovici, 2000 cited in Jodelet, 2003, p. 47) representing

“a system of interpretations and meanings tied to topics and events experienced within specific sociocultural environments. These representations are perceived and internalized by social actors and embedded into their broader cultural frameworks, ultimately guiding their understanding of the world.” (Abdelghani, 2017, p. 115)

These social representations shape how members of the study community understand illness and the role of frankincense, guiding their behaviors and choices regarding health and protection. Several participants also expressed that frankincense-based healing is a cultural heritage that must be preserved and transmitted across generations:

“*I raise my children on the scent of frankincense—it's the scent of our fathers and grandfathers.” —* S. Sh. (64 years), personal interview, Sohar, Sunday, 1 January 2023.

These findings demonstrate that frankincense-based healing practices in the studied community do not constitute residual or marginal traditions, but rather form part of a dynamic and enduring therapeutic system. Their persistence is sustained by shared cultural representations, ritual practices, and collective meanings that continue to shape how individuals understand and respond to illness. Importantly, this study does not seek to establish the biological efficacy of these practices, but rather to interpret their functions and meanings within the sociocultural context in which they are embedded.

## 5 Conclusion

This study explored the practices of healing and protection involving frankincense, considering it a prominent feature of traditional medicine in northern Al-Batinah, Oman, and an integral part of the community's customs and traditions. We approached the subject from various angles, grounded in fieldwork that provided the necessary data through observation and a series of interviews.

We began by tracing the roots of therapeutic and protective practices inspired by folk heritage and centered on frankincense, examining their ecological, historical, and cultural references. We found that healing practices involving frankincense are deeply linked to the Omani environment and its ecological characteristics. These practices are not only ancient but also deeply embedded in the historical and cultural structures of Omani society. Moreover, we concluded that the community's therapeutic behavior is shaped by its surrounding ecological environment, particularly the prominence of the frankincense tree and its long-standing historical and cultural uses in Oman. This relationship plays a significant role in shaping individuals' choices of healing and protective methods, favoring frankincense over other alternative remedies. As a result, these practices continue across various segments of the sample population, regardless of their social class or educational background.

The data also revealed the coexistence of two parallel therapeutic systems within the community:

- A traditional model, represented by widespread folk medicine practices—still actively used despite the development of modern healthcare systems.
- A modern model, backed by technology, laboratories, and scientific studies that have explored and suggested potential pharmacological properties of frankincense, particularly in relation to inflammation, rather than definitively establishing its clinical effectiveness.

These practices also exposed us to the representational systems held by the study's participants. Like other communities, they harbor specific beliefs about illness, its causes, and appropriate treatments. These representations reflect how people perceive their world—a world they believe to be shared with both visible and invisible forces. They see themselves as vulnerable to harm from both natural agents like viruses and bacteria, and supernatural entities such as the evil eye, envy, or jinn. In either case, frankincense is viewed by participants as both a healing and protective agent, whether used for respiratory infections like COVID-19 or for spiritual afflictions such as the evil eye or malevolent spirits.

Our inquiry into the practices involving frankincense revealed a wide range of forms and functions, confirming prior studies that emphasize the richness and diversity of Oman's medical history. This richness stems from Oman's geographic location, political history, and the influence of Indian, Islamic, Persian, Arab, and African healing traditions—as well as Portuguese medical approaches.

Furthermore, our findings confirm that frankincense-based healing and protective practices are ritualistic in nature, characterized by regularity, shared symbolism, codified performance, and perceived efficacy within the cultural context of the study community. Their attributed effectiveness, as expressed by participants, is rooted in three core sources:

- Cultural Continuity: Trust in ancestral knowledge and loyalty to traditions passed down through generations. These practices are performed as acts of reverence and emulation of forebears.
- Spiritual Belief: A sacred regard for the frankincense tree and a strong belief in the mystical power of its resin to affect individuals and communities positively.
- Scientific Interest: Support from contemporary laboratory research and academic studies—both within and beyond Oman—that indicate potential biological and pharmacological properties of frankincense extracts, particularly in relation to inflammation (Huang et al., 2022), without constituting direct clinical validation of its therapeutic efficacy in the contexts described in this study.

This convergence of cultural, spiritual, and scientific interest helps explain the continued presence and integration of frankincense-based practices within both formal and informal health contexts in Oman and beyond. This study does not aim to evaluate the clinical or biomedical efficacy of frankincense, but rather to understand its uses, meanings, and perceived effectiveness within the sociocultural context of the study community.

## Data Availability

The datasets generated during the current study are not publicly available due to ethical and confidentiality considerations but are available from the corresponding author on reasonable request.
